# Demographic costs of inbreeding revealed by sex-specific genetic rescue effects

**DOI:** 10.1186/1471-2148-9-289

**Published:** 2009-12-10

**Authors:** Susanne RK Zajitschek, Felix Zajitschek, Robert C Brooks

**Affiliations:** 1 Evolution & Ecology Research Centre and School of Biological, Earth and Environmental Sciences, University of New South Wales, Sydney, New South Wales 2052, Australia; 2 Station d'Ecologie Expérimentale du CNRS à Moulis, USR 2936, 09200 Moulis, France

## Abstract

**Background:**

Inbreeding can slow population growth and elevate extinction risk. A small number of unrelated immigrants to an inbred population can substantially reduce inbreeding and improve fitness, but little attention has been paid to the sex-specific effects of immigrants on such "genetic rescue". We conducted two subsequent experiments to investigate demographic consequences of inbreeding and genetic rescue in guppies.

**Results:**

Populations established from pairs of full siblings that were descended either from two generations of full-sibling inbreeding or unrelated outbred guppies did not grow at different rates initially, but when the first generation offspring started breeding, outbred-founded populations grew more slowly than inbred-founded populations. In a second experiment, adding two outbred males to the inbred populations resulted in significantly faster population growth than in control populations where no immigrants were added. Adding females resulted in growth at a rate intermediate to the control and male-immigrant treatments.

**Conclusion:**

The slower growth of the outbred-founded than inbred-founded populations is the opposite of what would be expected under inbreeding depression unless many deleterious recessive alleles had already been selectively purged in the inbreeding that preceded the start of the experiment, and that significant inbreeding depression occurred when the first generation offspring in outbred-founded populations started to inbreed. The second experiment revealed strong inbreeding depression in the inbred founded populations, despite the apparent lack thereof in these populations earlier on. Moreover, the fact that the addition of male immigrants resulted in the highest levels of population growth suggests that sex-specific genetic rescue may occur in promiscuous species, with male rescue resulting in higher levels of outbreeding than female rescue.

## Background

Inbreeding has detrimental effects on individual fitness in the wild [1] and in captive-bred populations [2]. This inbreeding depression is often manifested as low offspring survival [3], reduced fecundity and fertility [4,5], or decreased resistance to diseases and parasites [6,7]. At the population level, inbreeding depression can slow population growth and increase extinction risk [8-10], particularly in small and fragmented populations [11,12], making it a phenomenon of particular concern to conservation biology [13].

The level of inbreeding depression in any population also depends on a multitude of genetic factors. These include the previous history of inbreeding, genetic diversity, and the genetic basis of traits affecting fitness [14]. Experimental evidence suggests that the level of ancestral inbreeding as well as the rate of current inbreeding influence the level of inbreeding depression observed in populations [15,16]. For example, low rates of ancestral inbreeding reduced the genetic load and inbreeding depression in *Drosophila melanogaster*, as recessive deleterious alleles were purged from the population via selection [17]. Even though these effects have been observed in inbred laboratory strains, the role of selection against deleterious alleles in the restoration of fitness in the wild has been questioned [1].

In many naturally outbreeding populations no inbreeding depression or avoidance of inbreeding has been detected [18,19], but whether this is an actual lack of negative effects of inbreeding on fitness, or due to other factors is hard to determine. For instance, benign environmental conditions can mask deleterious effects [20]. Also, inbreeding and its deleterious effects can be severely underestimated in species that suffer most from inbreeding depression, if only the most heterozygous individuals survive when lethal alleles are expressed early in life [1]. The lack of suitable outbred control groups to evaluate the level of fitness losses can lead to the conclusion that the population under investigation is not negatively affected by inbreeding [14]. This scenario might occur especially when looking at rare and endangered populations, which are often already severely inbred, and for which outbred control populations do not exist anymore.

Adding unrelated individuals to populations suffering from inbreeding depression can immediately and dramatically increase heterozygosity and lead to an immediate improvement in fitness-related traits. This is known as genetic rescue [21-23]. In such cases, outbreeding leads to the masking of deleterious recessive alleles and to heterosis (hybrid vigour due to overdominance), and the positive effects on population growth rate increases the population size and reduces the possibility of sib-mating, thus reducing extinction risk [14,21]. Introducing immigrants into small, partially inbred populations has helped to improve reproductive fitness and greatly increased population size of adders [*Vipera berus*, [22,24]], and helped to increase egg viability in prairie chickens [*Tympanuchus cupido*, [23]]. Experimental addition of immigrants to an inbred natural population of Bighorn sheep led to large improvements of survival and reproduction [25]. A single immigrant has improved fitness by restoring population growth in a severely inbred population of grey wolves [*Canis lupus*, [26]]. In a *Daphnia magna *metapopulation, the fitness advantages due to hybrid vigour after experimental addition of immigrants were amplified, so that after multiple asexual generations followed by outbreeding, hybrid fitness was up to 35 times greater than that of residents [27].

Even though the benefits of immigration and translocation to small and inbred populations have been widely recognized among conservation biologists, little attention has been paid so far to sex-differences in the benefits that the immigrants might confer [but see [28]]. It has been hypothesised that female immigrants might particularly benefit small populations due to fitness increases by introducing new mitochondrial DNA, as mitochondrial mutations can negatively affect male fertility and thereby population viability [29,30]. Also, in systems where dominance hierarchies and infanticide are common, immigrant males might have negative impacts on population size by killing the existing juveniles [13,21]. For example, negative demographic effects of male immigration have been reported in the context of trophy hunting in brown bears [*Ursus arctos*, [31]] and lions [*Panthera leo*, [32]], and this was an important consideration in the relocation of panthers (*Felis concolor*) from Texas to Florida [33]. On the other hand, in the wide range of species where territorial and infanticidal aggression are low and mating with multiple males is common, translocating males instead of females should have much faster and greater effects on population growth and expected persistence time. In polyandrous species, male immigrants are able to inseminate and immediately produce outbred offspring with large numbers of females. Female immigrants, however, are limited by the number of offspring they are able to produce within a given breeding cycle, and are therefore more constrained than males in the immediate effects they can have on genetic rescue.

Natural guppy populations occur in pools separated by riffles and waterfalls in rivers and drainages in Trinidad [34]. Even though the separating riffles and waterfall barriers do not prevent gene flow [35], pools may be cut off from the connecting water bodies during the dry season [34]. This may genetically isolate the populations and may increase the danger of inbreeding in small pools, when reproductive effort is maximised during this season [34]. So far, no studies regarding population growth under inbreeding conditions have been undertaken in guppies. We investigate the effect of inbreeding on population growth in replicate experimental populations, each founded by one male and one female guppy (either a pair of inbred siblings or a pair of unrelated individuals) in order to understand the demographic effects of inbreeding. We then introduce either outbred males or outbred females to the inbred-founded populations to test experimentally for sex-specific effectiveness of immigrants in restoring fitness via genetic rescue. We test the prediction that introducing males to an inbred population will more effectively relieve inbreeding depression than introducing females.

## Methods

### Experiment 1: Measuring the demographic costs of inbreeding

The guppy population used for the experiments described in this study are descendents of animals captured in April 2002 from Alligator Creek, approximately 30 km south of Townsville (QLD, Australia). Guppies were introduced to Australia approximately 100 years ago, and the study population was most likely founded by animals from Guyana [36]. The initially N>1000 wild-caught adult males and females were kept as laboratory stock in ten to fifteen tanks each containing about one hundred fish. We moved animals between tanks monthly to ensure a well-mixed stock population. After approximately three generations under this regime in the laboratory, we established a set of 26 patrilines and kept both iteratively inbred and outbred animals within each patriline via a series of controlled matings. We used F3 descendants from these matings to start the current study. When we say two animals were unrelated, we mean that they most likely had no greater kinship than two randomly caught animals from the wild.

**Figure 1:**
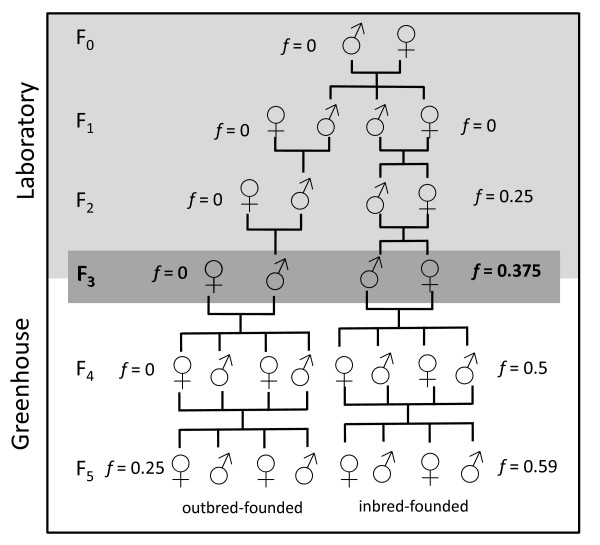
**Breeding design for inbred-founded (right hand side) and outbred-founded (left hand side) populations from a given patriline**. The light grey shaded area represents the part of the pedigree established under laboratory conditions to provide animals for this study. Laboratory-raised third generation animals (F3, dark gray shading) from seven patrilines were used as founders for the greenhouse populations. The unshaded area illustrates the two generations of offspring followed in Experiment 1.

We set up third generation offspring from paired inbred and outbred patrilines under semi-natural conditions (founders are F3 offspring in Figure 1). In a greenhouse, 14 plastic tubs holding 165 litres of water each and containing plastic plants and stone blocks (for spatial heterogeneity), were initially populated with a single male-female pair. This experiment was conducted in the UNSW greenhouse between December 2004 and May 2006. The greenhouse temperature was controlled not to drop below 15°C and not to rise above 30°C (on average 20.6°C ± 1.6 SD). Light was not supplemented or masked and so daylight cycles and light intensity followed those for Sydney, NSW (latitude -33.9° S).

Animals were fed fresh brine shrimp solution 3 times per week, and growing algae provided additional nutrients. To minimise the effects of parents breeding with offspring, but encourage sibling mating, the parental animals (easily identified by body size) were removed when the first offspring generation reached sexual maturity (aged approximately 4 months).

We set up seven replicate tanks of each of two experimental treatments. Each replicate of the **inbred-founded **treatment was established with a pair of full siblings that were descended from two generations of full-sibling inbreeding (Figure 1). They therefore each had an inbreeding coefficient (*f*) of 0.375 and a kinship coefficient (to one another) of *k *= 0.5. Each replicate of the **outbred-founded **treatment was established from an outbred, unrelated male-female pair (*f *= 0, *k *= 0; Figure 1, outbred). Once a month all fish were caught and counted. We used total number of fish per census as population size for each tank.

### Experiment 2: Rescue effects on experimental population growth

**Figure 2:**
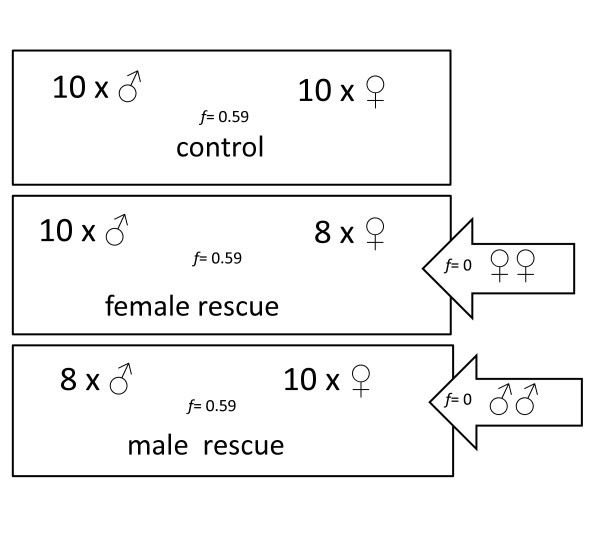
**Design of Experiment 2, testing for sex-specific rescue effects on population growth**. Fifth generation inbred offspring from inbred-founded replicates from Experiment 1 and outbred laboratory stock animals were used to stock the experimental populations (F5, *f *= 0.59, see Figure 1). All treatment groups had equal sex-ratios (1:1), and the populations were started with 20 individuals each. The control and female rescue treatments consisted of N = 4 replicates each, the male rescue of N = 3 replicates, due to abnormal mortality in one of the replicates. This design mimics translocation of outbred animals of one sex into a small, highly inbred population.

After 16 months, animals from four of the inbred-founded populations were split into same-sex tanks to avoid further uncontrolled inbreeding, and immature animals were raised to maturity. These animals had inbreeding coefficients of 0.59. In order to apply all treatments to each population and to test sex-specific effects of genetic rescue, we split each of the four inbred populations into three sub-samples (see Figure 2). The **control **treatment consisted of ten males and ten females from the same inbred population per replicate. The **female rescue **treatment consisted of eight inbred females and ten inbred male relatives plus two virgin females from laboratory outbred stocks. The **male rescue **treatment was established with ten females and eight males from the same inbred population plus two unrelated males from laboratory guppy stocks. All inbred females had been kept without males for a minimum of eight weeks to ensure female sexual receptivity, and that offspring were sired by the assigned males. During the first month of the experiments, survival was checked on a daily basis to ensure adequate founder numbers in the respective treatments. Within two weeks of the onset of the experiment, nine out of the ten males in one of the male-manipulated treatments died, and not enough animals were available to replace this replicate. It therefore was excluded from analyses. Animals in the remaining replicates were censused every three weeks to examine short-term differences in population growth, for a total of four months (July - October 2006, mean temperature = 19.8°C ± 1.6 SD).

### Statistical analysis

We analysed experimental population growth in both experiments 1 and 2 by fitting Linear Mixed Models with a Gaussian error distribution (command MIXED) in SPSS 15.0, using the monthly (experiment 1) or the 3-weekly (experiment 2) population count of each tank as a repeated measure. We fitted treatment and patriline, their interaction, and time of measurement as fixed effects, where treatment distinguished between inbred and outbred founders for experiment 1, and between control, female rescue, male rescue for experiment 2. We specified time of measurement also as a repeated effect. A first-order autoregressive covariance structure with equal variances (AR1) for the repeated effect provided the best fit when compared against other covariance structures (first-order autoregressive covariance structure with unequal variances, AR1H; first-order autoregressive moving average, ARMA(1,1); compound symmetry, CS) using Akaike's Information Criterion. The AR1 covariance structure is frequently used to fit models to longitudinal data with equally spaced observations and models higher correlations between observations that are closer together in time than between observations that are further apart in time [37].

## Results

### Population growth

**Figure 3:**
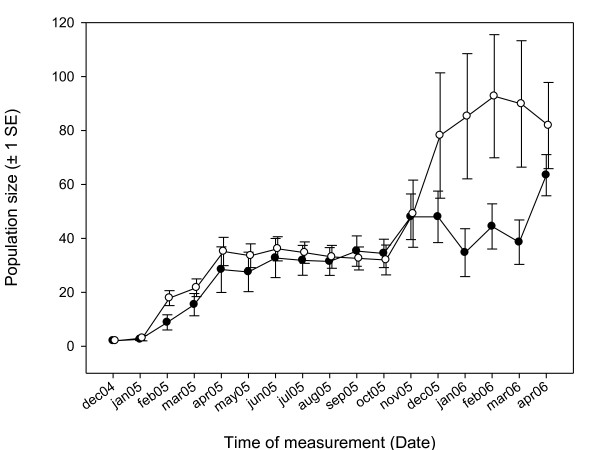
**Population growth for inbred-founded (open circles) and outbred-founded (black circles) treatments**. Shown are treatment means and error bars (± 1 SE) for tanks within treatments. The time between April and October marks winter 2005, where no population growth was occurring. Before then, first generation offspring were born (*f *= 0 in outbred-founded treatment, *f *= 0.5 in inbred-founded treatment). Second generation offspring (*f *= 0.25 in outbred-founded treatment, *f *= 0.59 in inbred-founded treatment) were born from October 2005 onwards. To help distinguish error bars, time of measurement for each treatment was changed by a small value to differ between treatments.

We found that population growth was not influenced by patriline (*F*_6,10 _= 0.40, *p *= 0.857) or treatment (*F*_1,10 _= 1.39, *p *= 0.267). The time of measurement and the treatment × time interaction were highly significant (monthly measurement: *F*_16,160 _= 6.78, *p *< 0.001, treatment × time: *F*_16,160 _= 3.36, *p *< 0.001), indicating that all populations were growing, and that there were time-dependent differences in growth rates in the inbred and outbred founded populations (see Figure 3). In general, population growth was affected by seasonal influences, as during the winter months (April-October 2006) population sizes remained steady.

### Sex-specific genetic rescue of inbred populations

**Figure 4:**
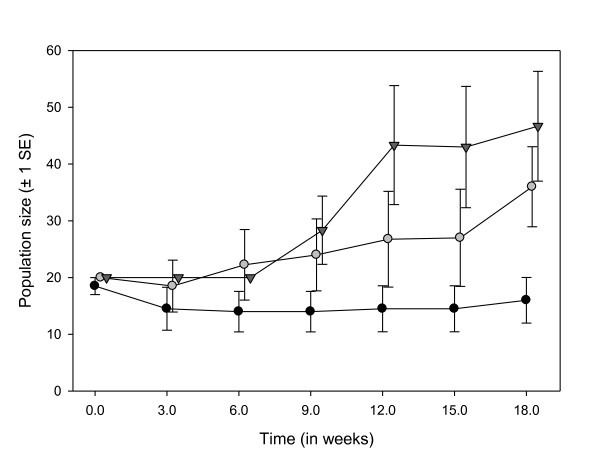
**Population growth in the three treatment groups**. Filled circles represent the control populations, where no outbred animals were added. Light grey circles represent the female-manipulated treatment and dark grey triangles represent the male-manipulated treatment. Points are treatment means, bars represent ± 1 standard errors. To help distinguish error bars, time of measurement for each treatment was changed by a small value to differ between treatments.

The treatment effect on population size is significant (*F*_2,7 _= 6.11, *p *= 0.032). Both week (*F*_6,43 _= 5.80, *p *< 0.001) and the interaction of treatment × week (*F*_12,43 _= 2.48, *p *= 0.015) are also significant, indicating changes in population size as well as treatment dependent differences in growth (Figure 4). Post-hoc pairwise comparisons showed that the male rescue treatment grew significantly faster than the control (*p *= 0.012), but that the female rescue treatment was not significantly different from the male rescue treatment (*p *= 0.144) or the control (*p *= 0.087). Nevertheless, it is possible that this marginal non-significance of the female rescue treatment is due to a lack of statistical power.

## Discussion

There were significant differences in population growth between the inbred- and outbred-founded experimental populations in experiment 1, mainly due to differences in growth rates that occurred after 13 months - that is in the second generation in the experimental tanks. Interestingly, the populations founded by inbred pairs strongly increased in numbers at this stage, whereas the outbred-founded treatment grew only modestly. By establishing populations from single male-female pairs, our measures of population growth in the first generation of this experiment may have lacked the power to detect differences between the inbred- and outbred-founded treatments. The seemingly counterintuitive faster growth in inbred-founded populations in the second generation might have been caused by greater inbreeding depression in the outbred-founded populations, experiencing their first generation of full-sibling inbreeding, than in the now heavily-inbred inbred-founded populations. This could occur if during the preparation and establishment of the first four generations of inbreeding the inbred patrilines had resulted in the selective loss of some deleterious recessive alleles affecting reproductive traits.

Selective purging of deleterious recessive alleles has been observed in laboratory experiments [17,38]. The efficiency of selection against deleterious recessive alleles in the reduction of the genetic load has been questioned, because the deleterious recessive alleles are usually masked from selection at low frequencies and their expression can be constrained by the environment in which they are expressed [7,39]. Experimental evidence suggests that, in small populations, selection can result in increased extinction risk rather than in a reduction of deleterious genetic material in a range of species [7,39-42]. Furthermore, it has been argued that selection against deleterious recessive alleles is most efficient when inbreeding occurs gradually [1,15]. The faster growth of highly inbred than newly inbred populations in our experiment indicates the potential for some selective purging of alleles involved with inbreeding depression with a rapid inbreeding via successive full-sib matings and in a competitive semi-natural environment.

The differences in population growth that inbred and outbred-founded populations showed in experiment 1 might, alternatively, occur by chance if there were little or no true inbreeding depression in this population of guppies. This seems unlikely, as we have found that sperm numbers were reduced in inbred individuals, and that highly inbred males are poor sperm competitors [43]. Further, the genetic rescue effects in our second experiment indicate that significant inbreeding depression was present within the inbred-founded populations. Strong inbreeding depression in the earliest stages of inbreeding is consistent with a behavioural study using first-generation inbred males from the same pedigree (*f *= 0.25), which found that display behaviour in inbred males also showed inbreeding depression. Inbred males courted less and spent less time following females compared to outbred males [44]. Similarly, populations from Trinidad suffer inbreeding depression in various fitness traits even under modest levels of inbreeding [45,46].

The results of our second experiment suggest there was indeed substantial inbreeding depression within the inbred-founded populations: adding outbred immigrants to populations of inbred full siblings resulted in a rapid increase in population growth. This genetic rescue effect has been observed in previous studies, in which immigration of outbred individuals led to increased population growth [22-26,47]. Such a rescue could be due to the masking of deleterious recessive alleles, particularly if purging underlying experiment 1 was only partial. Further, any increase in reproduction due to elevated heterozygosity and the presence of overdominance would also contribute to the observed result. The relative contributions of mutational load (deleterious recessives) and segregational genetic load (overdominance) in inbreeding depression in this system is a question ripe for further study, particularly because there is only very limited evidence that overdominance may contribute significantly to inbreeding depression in life history traits [48,49].

The increase in population growth was more pronounced in populations to which outbred males were introduced than those to which females were introduced. This makes sense in light of the promiscuous mating system of guppies, because introduced males would have been able to inseminate all available females leading to the potential for all females to outbreed within the first reproductive cycle. Previous experiments indicate that females prefer outbred to inbred males (SZ, unpublished data). Furthermore, outbreeding may have been facilitated by the well-known preference that female guppies have for males with rare colour patterns [50-52] as the outbred introduced males would have each had unique colour patterns compared with the inbred males who shared the same or very similar colour patterns (at a frequency of 0.8). Further, outbreeding may have been facilitated by superior sperm competitive ability of outbred males. We have shown using artificial insemination [43], that the sperm of highly inbred males is not as successful as that of outbred males in direct sperm competition. Despite all of these putative advantages to outbred males in mating with and fertilising females in the male-added treatment, the increase in population size depended on improved female fecundity and/or juvenile survival as a direct consequence of outbreeding.

Adding outbred females also increased population growth, but at a slower rate. This result is consistent with the hypothesis that outbreeding confers a fitness advantage, but only for matings that involve two outbred females in the females added treatment. Female reproductive output is limited by the number of eggs they can produce within a given breeding cycle. Because we only added two individuals, increased reproductive output in the first generation depended on these individuals compared to potentially the full complement of ten reproducing females in the male-manipulated treatment. Male immigrants were more successful in restoring fitness in the short term, and may be more efficient in restoring genetic variability in polyandrous species.

To date, little attention has been paid to the sex-specific value that immigrant or translocated animals have to small and inbred populations. So far, female immigrants have been valued for their introduction of new extra-nuclear genetic material [mitochondrial DNA, [29,30]]. Female immigrants are also favoured in species with strong hierarchical dominance systems, where immigrant males might have detrimental effects on population structure due to infanticide of resident juveniles [31,32]. Nevertheless, the value of male immigrants may have been underestimated in species with suitable polyandrous mating systems: our results suggest that males can restore fitness more efficiently than females in the short term, compared to purely inbred populations. Additional long-term studies will be needed to identify multiple generation effects of immigrants of both sexes, and to confirm the high value of male translocation to genetically rescue inbred populations for conservation biology.

In the guppy, males rather than females tend to disperse [53], but so far the extent and importance of sex-biased dispersal has been examined in few studies only [53,54]. Populations may be genetically isolated during the dry season [34], potentially leading to inbreeding and resulting in inbreeding depression. Immigrant males may help to genetically recover population growth not only by potentially outcompeting inbred males in sperm competition [43], but also due to female preferences for males with novel [50,51,55,56] or rare coloration patterns. Such mating preferences can result in highly increased mating success for immigrant males and might explain both male-biased dispersal and the levels of gene flow observed under natural conditions [35], even under conditions with relatively little dispersal.

## Conclusion

We have shown here that inbreeding affects population growth, and that this effect can be underestimated or overlooked. The high degree of inbreeding depression was only revealed by the addition of immigrants, as before that the inbred populations appeared to display normal growth rates following seasonal patterns. This has important consequences for the management of small populations, where an apparent absence of inbreeding depression might lead to the conclusion that no inbreeding is occurring. Moreover, adding male immigrants to small and fractured populations can lead to immediate fitness benefits, at least in promiscuous species.

## Authors' contributions

SZ drafted the manuscript and was the primary investigator of the experiments conducted. FZ helped with the experimental procedures, the statistical analyses and the writing of the paper. RB gave advice on the experimental design and helped with analyses as well as during the writing process. All authors read and approved the final manuscript.
